# Fownes’s Chemistry for Students

**Published:** 1845-09

**Authors:** 


### FOWNES' CHEMISTRY FOR STUDENTS.

The admirable prize essay of this author on "Chemistry as exemplifying the wisdom and beneficence of God," prepared us to expect a work of superior excellence on the general subject of chemistry. The only particular in which we have been disappointed is, that he has succeeded in comprising the matter of his work in 460 duodecimo pages, which, assuredly, is a recommendation of the volume as a text-baok for students. In this respect it has advantages over any treatise which has yet been offcred to American students. The difficulty in a text-book of chemistry is to treat the subject with sufficient fulness without going too much into detail. For students comparatively ignorant of chemical science, the larger systems are unprofitable companions in their attendance upon lectures. They need a work of a more elementary character, by which they may be inducted into the first principles of the science, and prepared for mastering its more abstruse subjects. Such a treatise is the one which we have now the pleasure of introducing to our readers; no manual of chemistry with which we have met comes so near meeting the wants of the beginner. All the prominent truths of the science, up to the present time, will be found given in it with the utmost practicable brevity. The style is admirable for its conciseness and clearness. Many wood-cuts are supplied, by which processes are made intelligible. The author expresses regret, that he could not enter more largely into organic chemistry, but his details will be found to embrace the most important facts in that interesting branch of the science. We shall recommend his manual to our class next winter. Y.

